# Supplementary material for: Oncology-Led Early Identification of Nutritional Risk: A Pragmatic, Evidence-Based Protocol (PRONTO)
Source: Cancers (Basel). 2023 Jan 6;15(2):380. doi: 10.3390/cancers15020380 (PMC9856655; doi:10.3390/cancers15020380)
Supplement: Supplementary file 1 [file cancers-15-00380-s001.zip › cancers-2061370-supplementary.pdf]

Supplementary information:[36-43]

Examples of tools to check for the presence of malnutrition and sarcopenia

**Tool**	**Description**
**Malnutrition**	
Canadian Nutrition Screening Tool[37] https://nutritioncareincanada.ca/sites/default/uploads/files/CNST.pdf	Based on two questions:<br>1. Have you lost weight in the past six months without trying to lose this weight?<br>2. Have you been eating less than usual for more than a week?
Global Leadership Initiative on Malnutrition (GLIM)[36]	A two-step approach:<br>1. Screening to identify "at risk" status by the use of any validated screening tool<br>2. Assessment for diagnosis and grading the severity of malnutrition
'Malnutritional Universal Screening Tool' https://www.bapen.org.uk/pdfs/must/must_full.pdf	Risk scoring system based on BMI, weight loss and acute disease effect (has been or likely to be no nutritional intake for >5 days)
MNA (Mini Nutritional Assessment) https://www.mna-elderly.com/	Based on six questions evaluating any decline in food intake, weight loss, mobility, psychological stress of acute disease, neuropsychological problems and BMI. Calf circumference may be used as an alternative to BMI
NRS-2002 (Nutritional risk screening score-2002) https://www.mdcalc.com/nutrition-risk-screening-2002-nrs-2002	Based on four questions:<br>1. BMI <20.5 kg/m^2<br>2. Weight loss within 3 months<br>3. Reduced dietary intake in the last week<br>4. ICU patient<br>If response to all questions is 'No', patient is considered low risk and at risk if 'Yes' to any.
**Sarcopenia / Strength**	
SARC-F*[38,41] https://www.cgakit.com/sarc-f-questionnaire	Based on five questions evaluating difficulty with strength (lifting and carrying), walking across a room), rising from a chair or bed and climbing 10 stairs and number of falls in the past year
SARC-CalF[42,43]	Consists of the 5 SARC-F questions with the addition of the measurement of calf circumference
Handgrip strength	A dynamometer is used to evaluate handgrip strength (average of 3 readings). Used in the diagnosis of sarcopenia
**Both malnutrition and sarcopenia**	
R-MAPP[39] https://www.clinicalnutritionjournal.com/article/S0261-5614(20)30222-3/fulltext	Combines three questions from the 'MUST' and the five SARC-F* questions
**Body composition**	
Computed tomography (CT) scan	Imaging tool for the measurement of body composition
Dual-energy X-ray absorptiometry (DXA)	Imaging tool for the measurement of body composition
Bioelectrical Impedance Analysis (BIA)	Measurement of body fat in relation to lean body mass by evaluating the impedance of a weak electric current

* is not useful for younger patients with cancer[26,40]

BMI, body mass index; ICU, intensive care unit; 'MUST', 'Malnutrition Universal Screening Tool'; R-MAPP, Remote Malnutrition APP; SARC-F, Strength, Assistance with walking Rise from a chair, Climb stairs and Falls.
